# A novel microfluidic chip-based sperm-sorting device constructed using design of experiment method

**DOI:** 10.1038/s41598-020-73841-3

**Published:** 2020-10-13

**Authors:** Chalinee Phiphattanaphiphop, Komgrit Leksakul, Rungrueang Phatthanakun, Trisadee Khamlor

**Affiliations:** 1 Degree Program in Industrial Engineering, Department of Industrial Engineering, Faculty of Engineering, Chiang Mai University, Chiang Mai, 50200 Thailand; 2 Industrial Engineering Department, Chiang Mai University, Chiang Mai, 50200 Thailand; 3 Synchrotron Light Research Institute (Public Organization), 111 University Avenue, Nakhon Ratchasima, 30000 Thailand; 4 Department of Animal and Aquatic Science, Faculty of Agriculture, Chiang Mai University, Chiang Mai, 50200 Thailand

**Keywords:** Nanoscale devices, Biomedical engineering

## Abstract

Microfluidics is proposed as a technique for efficient sperm sorting, to achieve the ultimate goal of resolving infertility problems in livestock industry. Our study aimed to design a microfluidic sperm-sorting device (SSD) through a high-efficacy and cost- and time-effective fabrication process, by using COMSOL Multiphysics simulation and modeling software, and the design of experiment (DOE) method. The eight factors affecting SSD performance were established. The simulation was then run, and statistically significant factors were analyzed. Minitab16 was used to optimize the design modulus factor. By setting the statistical significance at *p* < 0.05, the factors affecting experimental structure were analyzed. At a desirability of 97.99, the optimal parameters for the microfluidic chip were: angle between sperm and medium inlet chambers (A = 43°), sperm inlet flow rate (B = 0.24 µL min^−1^), medium inlet flow rate (C = 0.34 µL min^−1^), and inlet and outlet chamber lengths (D = 5000 µm). These optima were then applied to microfluidics device construction. The device was produced using soft lithographic microfabrication techniques and tested on Holstein–Friesian bull sperm. The highest bull sperm-sorting performance for this microfluidic device prototype was 96%. The error between the simulation and the actual microfluidic device was 2.72%. Fluid viscosity ranges analysis-based simulations revealed acceptable fluid viscosity tolerances for the SSD. The simulation results revealed that the acceptable tolerance range for fluid viscosity was 0.00001–0.003 kg m^−1^ s^−1^. This optimally designed microfluidic chip-based SSD may be integrated into sperm x/y separation micro devices.

## Introduction

Embryo survival determines ruminant milk and meat production yield, efficiency, and profitability. In Brazil, the estimated cattle embryo death rate has resulted in an annual loss of USD 350–850 million^1^. Assisted reproductive technologies such as in vitro fertilization (IVF) and intracytoplasmic sperm injection (ICSI) may be efficacious in solving certain infertility problems. IVF consists of mixing egg and sperm in a Petri dish and injecting the suspension into the uterus, while ICSI is a specialized IVF procedure wherein a single sperm is injected directly into the egg with a fine needle. Both these techniques have enhanced fertilization rates and are industry standards for infertility management in the livestock industry^2,3^.

In conventional semen analysis, quality is evaluated based on sperm count, motility, morphology, sperm plasma, and acrosomal membrane integrity. Although sperm quality plays a key role in chromatin modification during embryogenesis, its quality is often not taken into account^4^. While healthy spermatozoa can fertilize oocytes both in vivo (ovary) and in vitro, anomalies such as apoptosis, embryo disintegration, and miscarriage may nonetheless occur during embryogenesis^5,6^. Commercial products used to sort bull sperm such as the density gradient preparations BoviPure and Percoll separate up to 66.67% and 64.17% of the motile and nonmotile/dead sperms, respectively^7^. Evaluation of density gradient preparations indicates that BoviPure is efficacious in sperm separation for bovine IVF. Most bull sperm sorting is performed using density gradient preparations. However, a sperm-sorting device (SSD) with microfluidic inserts may be more accurate and reliable than density gradient preparations. Moreover, it is compact, easy to use, decreases the chances of human error, accelerates response time and diagnosis, requires reduced sample volumes, and is cost-effective. SSDs may also be used to develop convenient sperm motility assays that may be used in the field at the point of care.

Microfluidic technologies have been the focal point for numerous types of biomedical research over the past decade. They facilitate the creation of various in vitro models that closely simulate mammalian microenvironments^8^. These technologies have been applied in the development of sorting systems for analysis of sperm morphology, motility, and DNA integrity^9^. Several studies have reported the development of sperm-sorting systems mainly by using microfluidics technology^10–13^. The most successful microfluidic sperm-sorting systems are passively driven. These were originally configured by using loaded sperm and medium input channels, and nonmotile and motile output channels^10–13^. A K-shaped, micro-scale, integrated sperm sorter design was proposed by Chung et al.^10^. Huang et al.^14^ proposed a version with four, rather than two output channels. However, there is empirical evidence that two exit channels more efficiently sorted sperms than other designs^14^. Therefore, we built upon prior research by investigating two exit channel-based sperm sorter designs. The COMSOL program was applied to optimize structural design. In the simulation process, sperm was assumed to be spherical in shape with zero velocity. Flow rate of semen with motile/nonmotile sperm and HEPES were controlled by a syringe pump. Mixing of two streamlines was maintained at a creeping flow pattern to obtain high sperm-sorting efficiency. To control creeping flow of mixed streamline, the sperm streamline is required to cover nearly 40% of the overall central or separation channel width. Computer simulations can help lower research and development costs especially at the design phase. More detailed and elaborate structures based on two entrance and two exit channels were explored. The required parameters were angle between chambers, flow rates at the sperm and medium inlets, inlet and outlet chambers lengths, separation chamber length, inlet and outlet chamber thicknesses, separation chamber width, and inlet and outlet chamber widths. Thus, the main objective of simulation was to search for an optimal design in which the sperm streamline covered nearly 40% of the overall separation channel (proportional to sperm streamline width: separation channel width), while the mixed fluid streamline followed creeping flow pattern. Healthy motile sperm in sperm streamline will swim across its streamline to find the nutrients in medium streamline within the residence time. Verification and validation of model were performed before conducting simulation-based design of experiment (DOE). Moreover, the most promising factors and optimal design had to be identified by statistical methods. The obtained optimal microfluidic chip was used in the fabrication process, and its efficacy was tested by sorting bull sperm. Finally, fluid viscosity ranges which effect the sorting efficiency were determined by simulations. The design thus developed will be helpful in achieving the ultimate goal of efficaciously resolving livestock infertility problems, by feasible integration of motile/nonmotile sperm sorting with sperm count and separation.

## Materials and methods

### Fluid flow simulation in microfluidic channels

**Figure 1:**
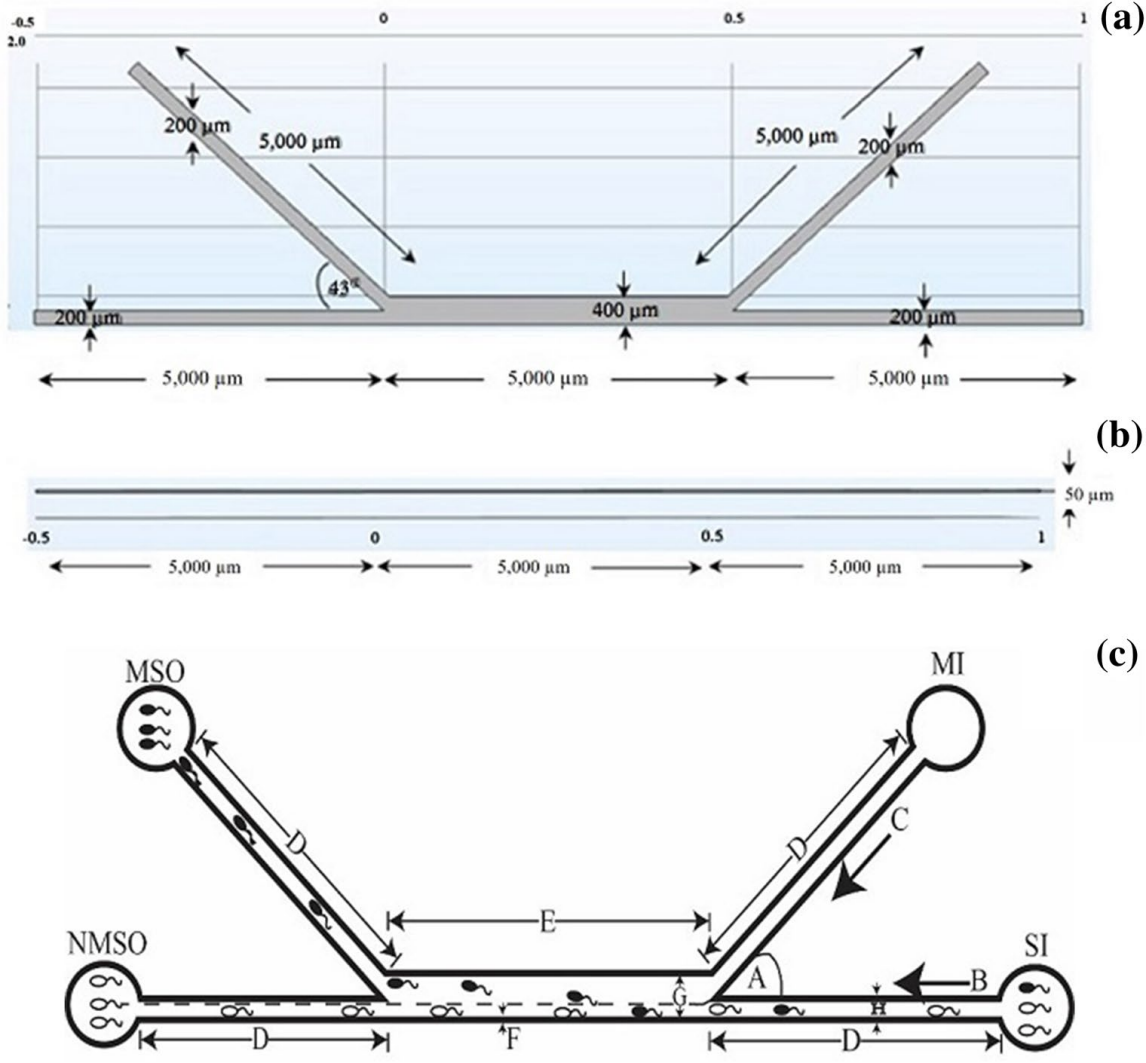
Geometry of the sperm-sorting device, related variables, and abbreviations. *MI* medium inlet, *SI* sperm inlet, *MSO* motile sperm outlet, *NMSO* non-motile sperm outlet, *A* angle between chambers, *B* sperm inlet flow rate, *C* medium inlet flow rate, *D* inlet and outlet chamber lengths, *E* separation chamber length, *F* inlet and outlet chamber thicknesses, *G* separation chamber width, *H* inlet and outlet chamber widths.

The COMSOL program is based on a Newtonian fluid flow assumption. Here, fluid in the separation channel is the mixed fluid with diluted semen and medium, with the expected flow rate ratio 1:1.5, and has very less viscosity, thus assuming conditions of a Newtonian fluid. The Reynolds number (Re) formula for fluid flow in small channels, supporting medium and sperm streams in close proximity to each other without turbulent mixing, can be calculated as:

1 $$ {\text{Re}} = \frac{\rho UD}{\mu } $$

where, *ρ* is the fluid density at a semen volume (*V*) of 0.2 × 10^−5^ m^3^, and the mass (*m*) is 0.001 kg. Thus, $\rho = \frac{{m\;({\text{kg}})}}{{V\;({\text{m}}^{3} )}} = \frac{0.001}{{0.000002}} = 500\;{\text{kg}}/{\text{m}}^{3}$. *U* is the characteristic flow velocity and is assigned as 2.5 × 10^−10^ mm s^−1^ (weighted sperm and medium inlet flow rate), D (characteristic device channel diameter) = 0.74 mm, and *μ* (fluid viscosity) = 0.00089 kg m^−1^ s^−1^ ^14,15^. Thus, Re = 1.039 × 10^−5^. When Re <  < 1, a creeping flow interface may be used. The chamber diffusivity was calculated with the Stokes–Einstein and Peclet number (*P*_*e*_) equations mentioned below:

2 $$ d = \frac{{k_{B} \times T}}{{\overline{f} }} $$

3 $$ P_{e} = \frac{LU}{d} $$

where, *k*_B_ is the Boltzmann’s constant (~ 1.38 × 10^−23^ J K^−1^), *T* is the absolute temperature (K), and $\overline{f}$ is the particle dimension and fluid viscosity. A spherical particle is defined by $\overline{f} = 6 \times \pi \times \mu \times R$, where *R* is the sphere radius. In our simulation approach, sperm was assumed to be spherical, with a diameter of ~ 6 µm^14^, while real sperm has an ellipsoid head (5 µm or 3 µm) with a large tail (30–50 µm). However, the spherical shape was reasonably assumed to generate greater drag force than that by an ellipsoid shape, and diameter ratio between head and tail is very large. The $d$ value was calculated according to Eq. (2) to determine *P*_*e*_. These $d$ values corresponded to *P*_*e*_ > 1, and implied that the cellular *P*_*e*_ was significantly > 1. Numerical stabilization is necessary to solve Fick’s equation, and COMSOL automatically stabilizes this by default without any explicit settings. The model set $d$ = 1.5 × 10^−13^ m^2^ s^−1^ for all COMSOL simulations. Density and viscosity were assumed to be constant. The former was considered to be equal to that of water at 20 °C, i.e., 9.98 × 10^−1^ mol m^−3^. Viscosity was set to 0.5 m^6^ mol^−2^. This relationship between concentration and viscosity is usually observed for solutions consisting of large molecules. Sperm and HEPES medium viscosities were assumed to be 0.00089 kg m^−1^ s^−1^ and 0.0007 kg m^−1^ s^−1^, respectively, at 28 °C^14^. According to our proposed method, geometry of the SSD, related variables, and parameter abbreviations have been shown in Fig. 1a–c. The boundary conditions for our simulation approach are as follows: diffusion constant = 1.5 × 10^−13^ m^2^ s^−1^, medium inlet flow rate = 0.2–0.45 µL min ^−1^, sperm inlet flow rate = 0.15–0.4 µL min^−1^, medium concentration = 0 mol m^−3^, sperm concentration = 0.988 mol m^−3^, and free quad mesh.

#### Verification of the simulation model

**Figure 2:**
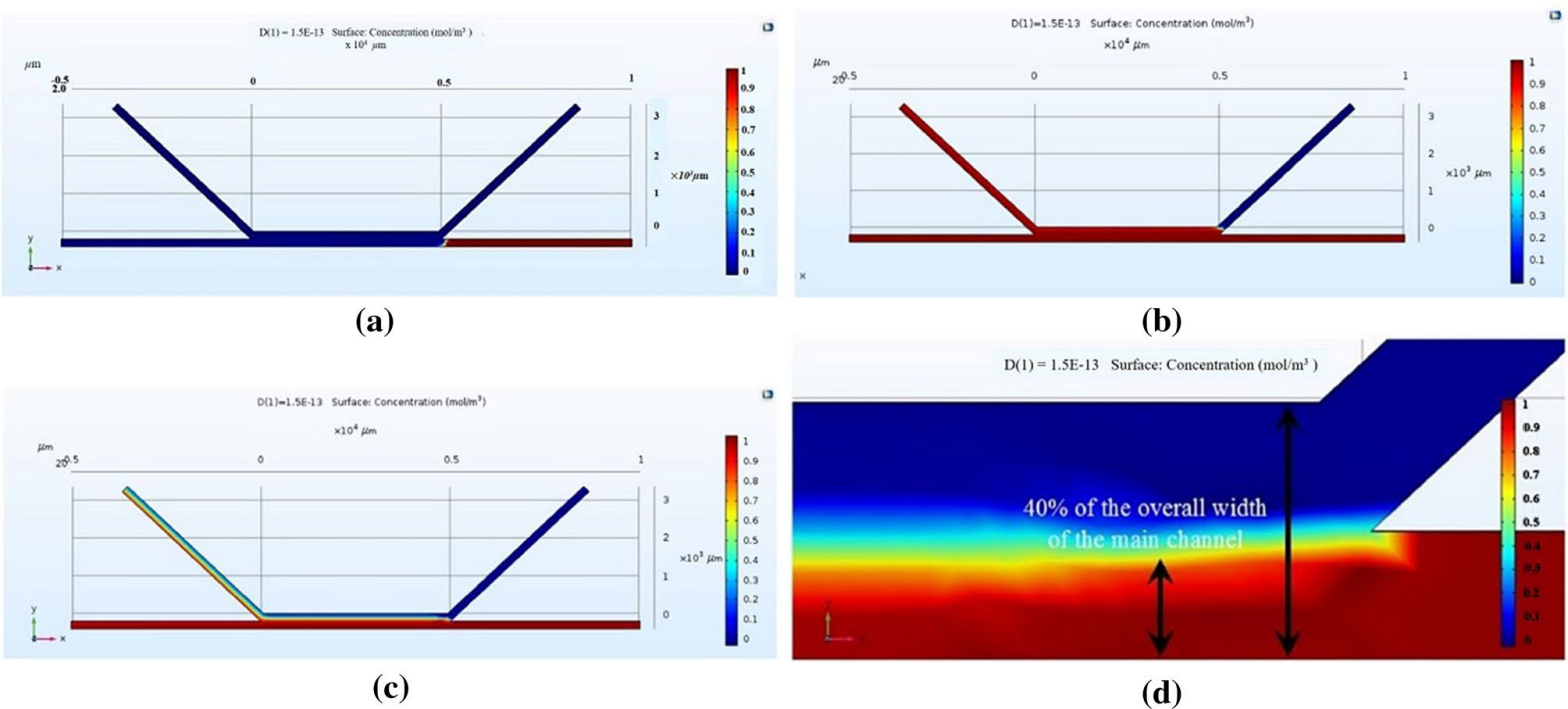
Model verification (**a**) high medium inlet flow rate, (**b**) high sperm inlet flow rate, (**c**) sperm flow rate reached over 40% of the separation channel width (**d**) enlarge of (**c**).

The simulation model was verified by varying medium and sperm flow rates. As per our expectation, sperm could not flow into the main channel upon increasing medium inlet flow rate (Fig. 2a), whereas medium could not flow into the main channel upon increasing sperm inlet flow rate (Fig. 2b). Also, we could notice the sperm streamline flow to upper outlet channel (Fig. 2c) when sperm flow rate reached over 40% of the separation channel width (Fig. 2d).

#### Validation of the simulation model

**Figure 3:**
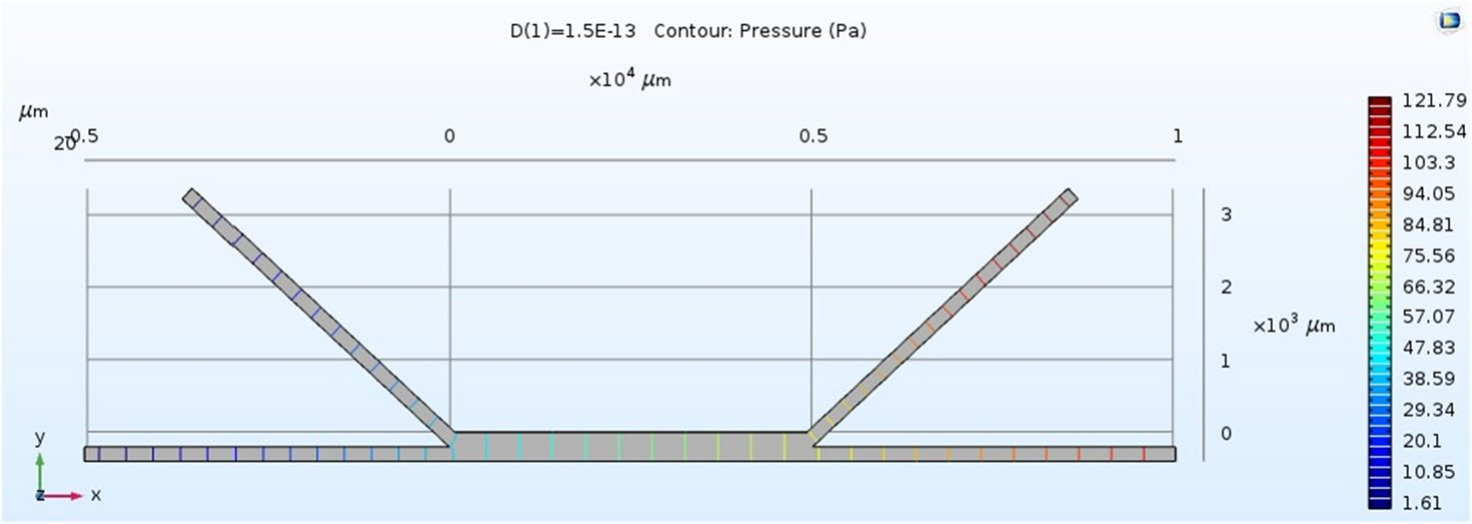
Pressure at two outlet positions from simulation.

We measured the pressure drop at motile sperm outlet (MSO) and nonmotile sperm outlet (NMSO), and compared them with the simulation results (Fig. 3). We found that actual pressure drops from the designed chip at two outlet positions (MSO and NMSO) were close to simulation results from the COMSOL program.

### Design of experiment (DOE) for the sperm-sorting device

Core parameters of the SSD were improved by a two-level factorial design analyzed in Minitab18. Multifactors were screened by a two-level (or 2^*k*^) factorial design to study the effects of the SSD. The interactions between creeping sperm flow sorting were analyzed at 40% of the overall central or separation channel width. The optimal values for the factors were used as response variables in the fabrication of the device, and the evaluation of its performance.

### Experimental design and statistical analysis

**Figure 4:**
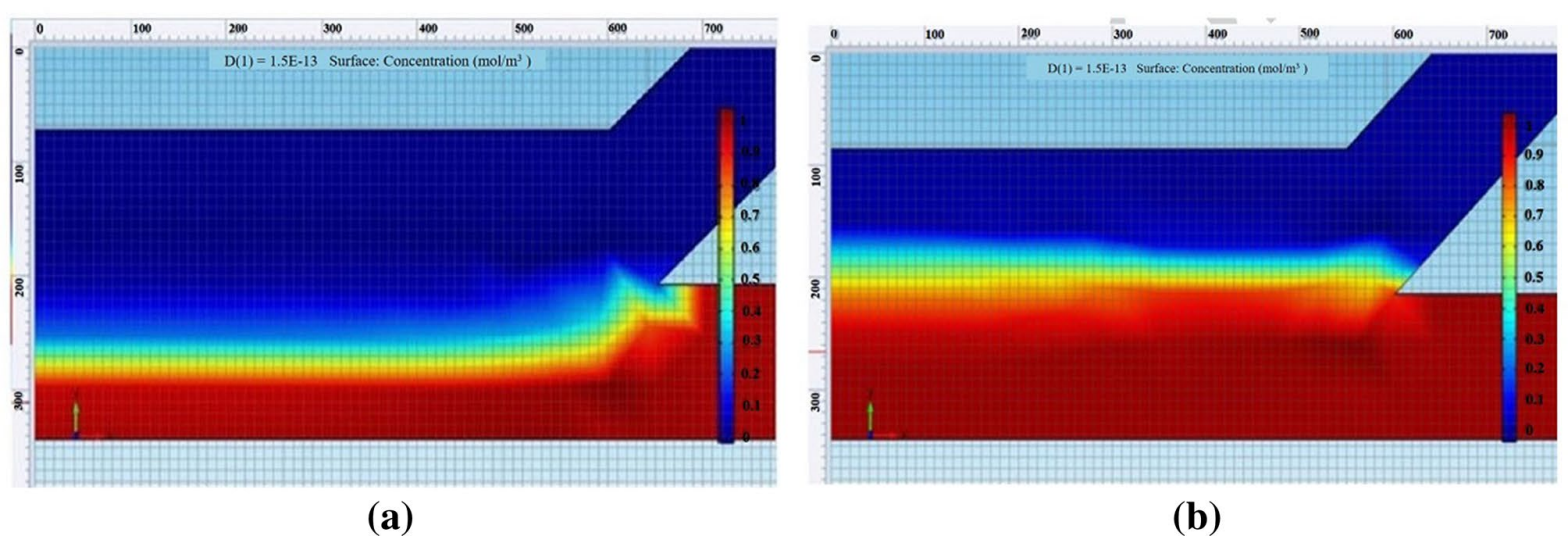
Grid count to obtain simulation of % of sperm streamline from overall separation channel width (**a**) lower grid count (**b**) higher grid count.

Based on the SSD structure (Fig. 1), the following eight parameters were evaluated: angle between chambers (A), sperm inlet flow rate SI (B), medium inlet flow rate MI (C), SI, MI, MSO, and NMSO lengths (D), separation chamber length (E), SI, MI, MSO, and NMSO thicknesses (F), separation chamber width (G), and SI, MI, MSO, and NMSO widths (H). Simulation experiments were conducted to obtain the low and high values for each factor at a sperm streamline of nearly 40% of the SSD main channel. A grid count (Fig. 4) was used to obtain the sperm streamline/main channel ratio. We could notice that sperm streamline (red) in Fig. 4a has a lesser grid count than Fig. 4b. By varying a single factor and fixing all others in each experiment, numerous prescreening simulation experiments were conducted considering a sperm streamline of nearly 40%. From the preliminary screening, we proposed a DOE to obtain an optimal of combined factors and interaction effects. Then, the low and high levels of each factor were obtained for the DOE settings (Table 1). According to these values, there were one-quarter of the total number of experimental sets $\left( {\frac{1}{4} \times 2^{8} } \right)$, or 64 runs (Supplementary Table B1) based on the DOE approach. A statistical analysis was performed to screen for the most useful factors^16–22^.

**Table 1:** Factors, levels, and symbols for sperm-sorting device.

Factor	Level Low (− 1)	Level High (+ 1)	Symbol
Angle between chambers (°)	33	43	A
Sperm inlet flow rate (µL min^−1^)	0.15	0.40	B
Medium inlet flow rate (µL min^−1^)	0.20	0.45	C
SI, MI, MSO, NMSO lengths (µm)	3000	5000	D
Separation chamber length (µm)	3000	5000	E
SI, MI, MSO, NMSO thicknesses (µm)	30	50	F
Separation chamber width (µm)	300	400	G
SI, MI, MSO, NMSO widths (µm)	100	200	H

*MI* medium inlet, *SI* sperm inlet, *MSO* motile sperm outlet, *NMSO* non-motile sperm outlet, *A* angle between chambers, *B* sperm inlet flow rate, *C* medium inlet flow rate, *D* inlet and outlet chamber lengths, *E* separation chamber length, *F* inlet and outlet chamber thicknesses, *G* separation chamber width, *H* inlet and outlet chamber widths.

### Device fabrication

The microfluidic device was designed with Layout Editor 2015 (juspertor GmbH, Unterhaching, Germany). The microchannel pattern was printed either with UV-opaque ink for plastic films, or with chromium for glass plates. The microfluidic mold was fabricated by soft photolithography, which uses light to transfer geometric patterns from a photomask to a photoresist onto the substrate. After the photoresist is exposed to UV via the photomask, fluidic channel mold micropatterns are made. The microfluidic mold was composed of SU-8 photoresist formed on a glass slide with a 50-μm flow channel depth. The flow channel substrate consisted of polydimethylsiloxane (PDMS) and was prepared by mixing the PDMS with the curing agent on the mold in a 10:1 ratio. Bubbles were removed by degassing. The substrate was baked at 60 °C for 1 h.

The microfluidic chip used for sperm sorting was fabricated by cleaning the glass slide, by patterning the SU-8 2100 layer for the microfluidic mold, and by creating the PDMS Sylgard-184 substrate based on the mold (Supplement material, Fig. A1). The microfluidic device inlets and outlets were punched with a needle or punch of the same size as the connection tubes. The PDMS surface was treated with oxygen plasma for 30 s and bonded to a glass slide to prevent fluid leakage. Polymer tubes were connected to the punched holes to deliver the solutions.

### Bull sperm preparation

Holstein Friesian bull semen samples were purchased from the Dairy Farming Promotion Organization of Thailand, Mittraphap Subdistrict, Muak Lek, Saraburi, Thailand. A single bull semen sample straw containing 30 × 10^6^ spermatozoa, preserved by deep-freezing in liquid nitrogen, was thawed in a water bath at 37 °C for 40 s. The thawed sample was then placed in a 1.5-mL microcentrifuge tube and stored in a chamber at a constant 37 °C. Live sperms with mortality > 70% were used. One microliter warm Beltsville-TS (BTS) extender at 37 °C was added to remove the egg yolk extender from the spermatozoa. Sperm sample tube was centrifuged at 5000 rpm for 5 min, and the supernatant was discarded to collect the sperm pellet. A final density of 3 × 10^6^ sperms/100 µL was prepared with warm BTS extender. To maintain sperm viability, the suspension was incubated in a chamber at a constant 37 °C.

### Fluorescence experiment

Motile sperm were stained green with SYBR-14 dye. Nonmotile sperm were stained red with propidium iodide. The live sperm in the semen sample fluoresced bright green at ~ 518 nm excitation. Ten microliters of pure SYBR-14 was added to the semen sample and the suspension was incubated for 15 min. Then propidium iodide was added to the suspension, and it was incubated for 5 min in a chamber at a constant 37 °C. The suspension was then washed thrice by centrifugation with 1000 µL extender for 7 min each time. The cleaned suspension was then viewed and photographed under a fluorescence microscope (Olympus IX71).

Motile sperm in channels SI and MSO were counted in a hemocytometer. The number of sperms in the inlet (Channel SI) and outlet (Channel MSO) were determined to calculate the % of high-motility sperm derived from the sorting device. The results of all experiments were assessed to establish whether they followed the patterns reported by the COMSOL simulation. For the creeping flow, the response was configured at 40% sperm streamline of the total structure width. DOE was used to isolate the optimized parameters for sorting sperm cells. The sperm cells were counted using Eq. (4) as follows:

4 $$ N\left( {\text{cells/mL}} \right) = \frac{{\alpha \times {\text{Dilution factor}} \times {400} \times {10}^{{3}} }}{9 \times 16 \times 0.1} $$

where, $\alpha$ is the number of sperm cells.

### Ethical approval

The semen samples analyzed in the present study were not directly obtained from cattle. The samples were provided by the Production Center of the Inthanon Royal Project, Agriculture Faculty, Chiang Mai University. The lot number was HF96TH362. The bull sperms were stored in long straws at 5 °C that were then immersed in liquid nitrogen at − 196 °C.

## Results and discussion

### Simulation program (COMSOL)

**Figure 5:**
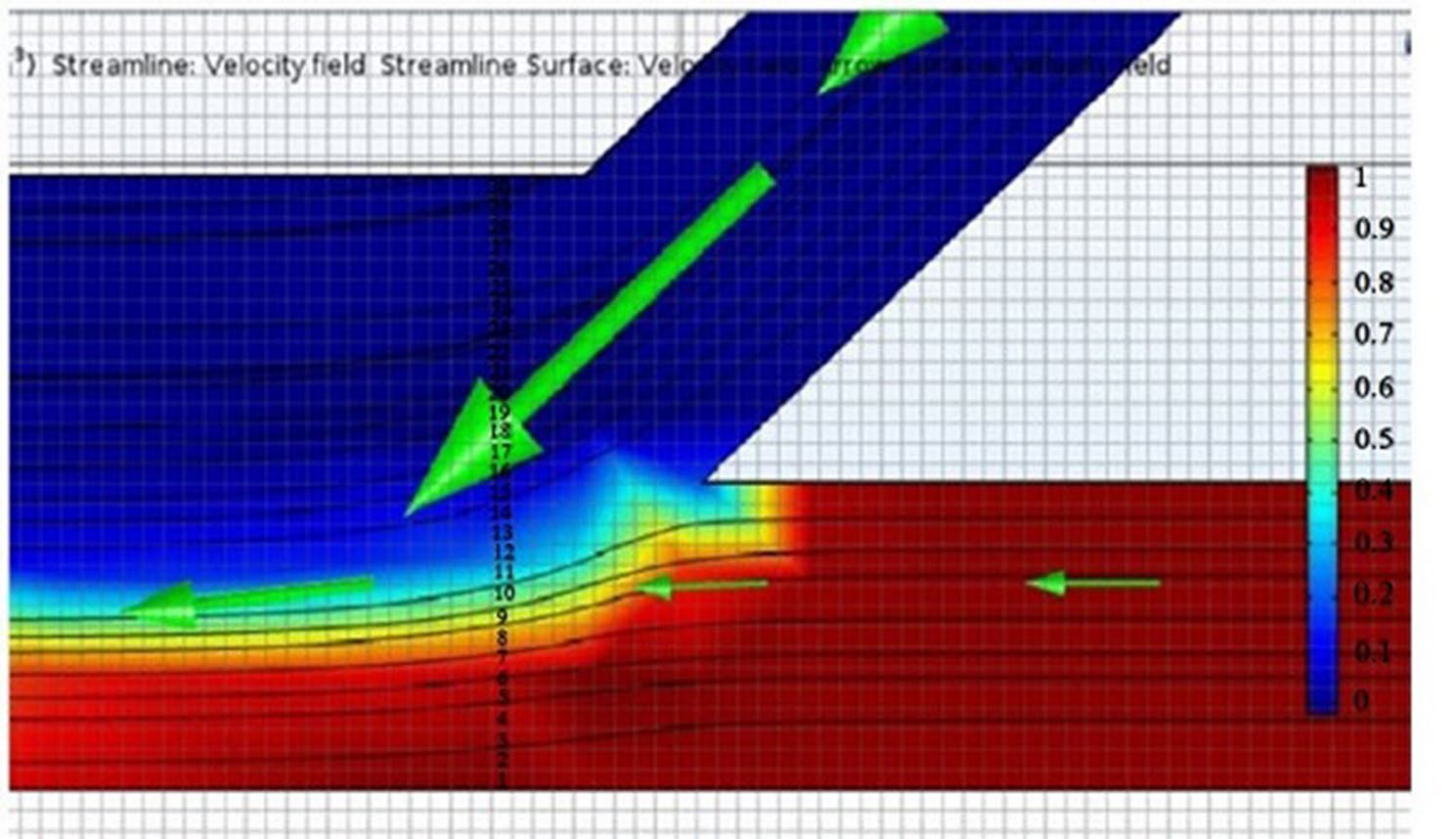
30% sperm streamline in the separation channel.

**Figure 6:**
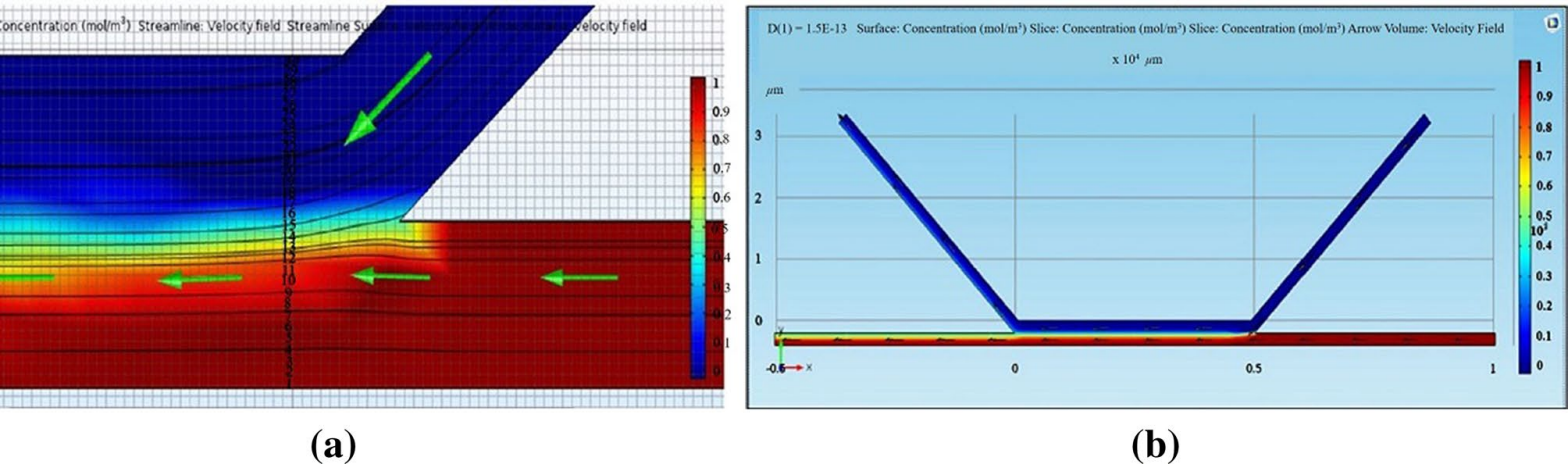
(**a**) 40% sperm streamline for overall width of main channel preformed creeping flow, derived from COMSOL simulation. (**b**) Structure of 40% sperm streamline for overall width of main channel.

The simulation COMSOL program was used to obtain a % sperm streamline and separation channel width. From the simulation (Fig. 5), we obtained nine sperm streamline pixels out of thirty pixels of total separation width (30%). The target range of sperm streamline concentration was 0.6–1.0. At 40% (12/30 pixels), the sperm streamline to separation channel and a 43° angle between the chambers provided smooth creeping flow (Fig. 6a,b). The % of sperm streamline responds to the efficiency of the SSD. As per the previous report^10^, 40% sperm streamline was suggested. Under high pressure from medium streamline (less than 40% sperm streamline) at constant flow rate, motile sperm could not swim well to the motile sperm outlet. Conversely, low pressure (more than 40% sperm streamline) caused the mixing of motile and non-motile sperm cells.

### Statistical analysis

The experiments were designed and conducted under 64 different conditions. The % of sperm streamline is presented in Supplementary Table B1. A pre-screening analysis of variance (ANOVA) identified the responses of the main factors affecting the SSD design. The data were generated on the basis of a significant statistical regression equation and described the relationship between the operational variables and the responses (*p* < 0.05). The statistical regression coefficient (R^2^) measured the linear model fit.

**Figure 7:**
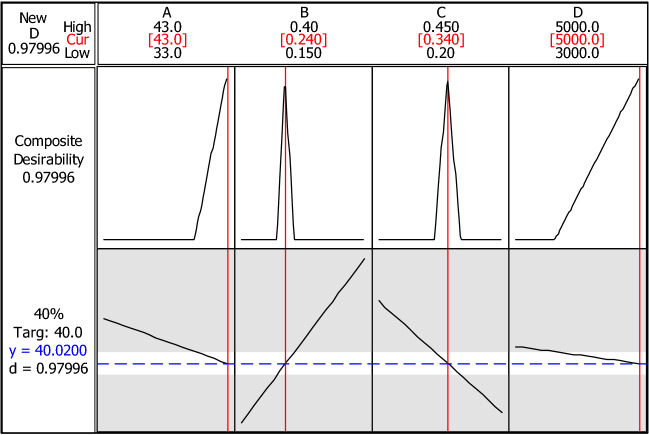
Optimal values for sperm-sorting device. Output shows that optimal values are A = 43, B = 0.24, C = 0.34, and D = 5000. Desirability = 0.97996. *A* angle between chambers, *B* sperm inlet flow rate, *C* medium inlet flow rate, *D* inlet and outlet chamber lengths.

Table 2 shows that the four main factors, viz., angle between the chambers (A), the sperm inlet flow rate (B), the medium inlet flow rate (C), and the inlet and outlet chamber lengths (D) are statistically significant. The screening experiment indicates that the aforementioned factors should be analyzed by a full factorial design (2^4^) with two replicates in 32 runs (Table 3), and by running a test to obtain the response in the COMSOL program. The output was analyzed by Minitab18 and an optimal SSD design was acquired. The regression models (Eq. (5)) described the relationships among A, B, C, and D, and their responses. The equations included linear and interrelated terms (Table 4). The R^2^ was adjusted to be > 99.89%, which was a high reliability score. Moreover, a statistical analysis was applied to optimize this model (Fig. 7). The optimal response values were: (A) angle between chambers = 43°; (B) sperm inlet flow rate = 0.24 µL min^−1^; (C) medium inlet flow rate = 0.34 µL min^−1^; and (D) inlet and outlet chamber lengths = 5000 µm. Separation chamber length (E), SI, MI, MSO, and NMSO thickness (F), separation chamber width (G), and SI, MI, MSO, and NMSO width (H) were not statistically significant, within their range defined in Table 1, as promising factors affecting the flow pattern and SSD performance. E and G, which are the main components, do not play a significant role in the performance because their defined range in our experiment are wide enough for generation of sperm swimming during residence time. ANOVA identified the significant factors and these, in turn, were used to formulate the following regression model (Eq. 5):

5 $$ {\text{Y}} = {44}.{921} - {3}.{\text{413A}} + {1}0.{\text{723 B}} - {15}.{\text{336C}} - {1}.{\text{234 D}} + {1}.0{\text{22AB}} - {3}.{\text{689BC}} + {1}.{4}0{\text{1BD}} + {2}.0{\text{33CD}} - {1}.{1}0{\text{6ABC}} + {1}.{\text{656ABD}} + {1}.{\text{495ACD}} - {2}.0{1}0{\text{BCD}} - {1}.{\text{682ABCD}} $$

**Table 2:** Regression analysis. Response at 40% of overall width of central or separation channel vs. A, B, C, D, E, F, G, and H.

Predictor	Coefficient	SE coefficient	T	P
Constant	78.08	4.90	15.92	0.000
A	− 0.8311	0.0739	− 11.25	0.000
B	44.45	2.95	15.04	0.000
C	− 50.43	2.95	− 17.07	0.000
D	0.001393	0.000369	3.77	0.000
E	− 0.000198	0.000369	− 0.54	0.593
F	− 0.0157	0.0369	− 0.43	0.672
G	0.00023	0.00739	0.03	0.975
H	0.00329	0.00739	0.45	0.658
S = 2.95458, R^2^ = 0.923, R^2^ (adj) = 0.9118				

*SE* Square error, *T* T-test, *P* P-value.

**Table 3:** Experimental design.

StdOrder	RunOrder	CenterPt	Blocks	A	B	C	D
4	1	1	1	43	0.4	0.2	3000
25	2	1	1	33	0.15	0.2	5000
13	3	1	1	33	0.15	0.45	5000
6	4	1	1	43	0.15	0.45	3000
3	5	1	1	33	0.4	0.2	3000
9	6	1	1	33	0.15	0.2	5000
32	7	1	1	43	0.4	0.45	5000
14	8	1	1	43	0.15	0.45	5000
21	9	1	1	33	0.15	0.45	3000
20	10	1	1	43	0.4	0.2	3000
27	11	1	1	33	0.4	0.2	5000
11	12	1	1	33	0.4	0.2	5000
17	13	1	1	33	0.15	0.2	3000
28	14	1	1	43	0.4	0.2	5000
5	15	1	1	33	0.15	0.45	3000
29	16	1	1	33	0.15	0.45	5000
24	17	1	1	43	0.4	0.45	3000
30	18	1	1	43	0.15	0.45	5000
7	19	1	1	33	0.4	0.45	3000
1	20	1	1	33	0.15	0.2	3000
2	21	1	1	43	0.15	0.2	3000
31	22	1	1	33	0.4	0.45	5000
23	23	1	1	33	0.4	0.45	3000
26	24	1	1	43	0.15	0.2	5000
12	25	1	1	43	0.4	0.2	5000
16	26	1	1	43	0.4	0.45	5000
15	27	1	1	33	0.4	0.45	5000
10	28	1	1	43	0.15	0.2	5000
18	29	1	1	43	0.15	0.2	3000
19	30	1	1	33	0.4	0.2	3000
22	31	1	1	43	0.15	0.45	3000
8	32	1	1	43	0.4	0.45	3000

2^4^ factorial design with two replicates.

*A* angle between chambers, *B* sperm inlet flow rate, *C* medium inlet flow rate, *D* inlet and outlet chamber lengths.

**Table 4:** Analysis of Minitab18 output.

Term	Effect	Coefficient	SE coefficient	T	P
Constant		44.921	0.06041	743.56	0.000
A	− 3.413	− 1.706	0.06041	− 28.24	0.000
B	10.723	5.361	0.06041	88.74	0.000
C	− 15.336	− 7.668	0.06041	− 126.93	0.000
D	− 1.234	− 0.617	0.06041	− 10.21	0.000
A*B	1.022	0.511	0.06041	8.46	0.000
A*C	0.076	0.038	0.06041	0.63	0.537
A*D	0.111	0.056	0.06041	0.92	0.371
B*C	− 3.689	− 1.844	0.06041	− 30.53	0.000
B*D	1.401	0.701	0.06041	11.6	0.000
C*D	2.033	1.016	0.06041	16.82	0.000
A*B*C	− 1.106	− 0.553	0.06041	− 9.16	0.000
A*B*D	1.656	0.828	0.06041	13.71	0.000
A*C*D	1.495	0.747	0.06041	12.37	0.000
B*C*D	− 2.010	− 1.005	0.06041	− 16.64	0.000
A*B*C*D	− 1.682	− 0.841	0.06041	− 13.92	0.000
S = 0.341751 PRESS = 7.4748					
R^2^ = 99.94%; R^2^ (pred) = 99.76%; R^2^ (adj) = 99.89%

### Sperm-sorting test on microfluidic chip using optimal parameters

**Figure 8:**
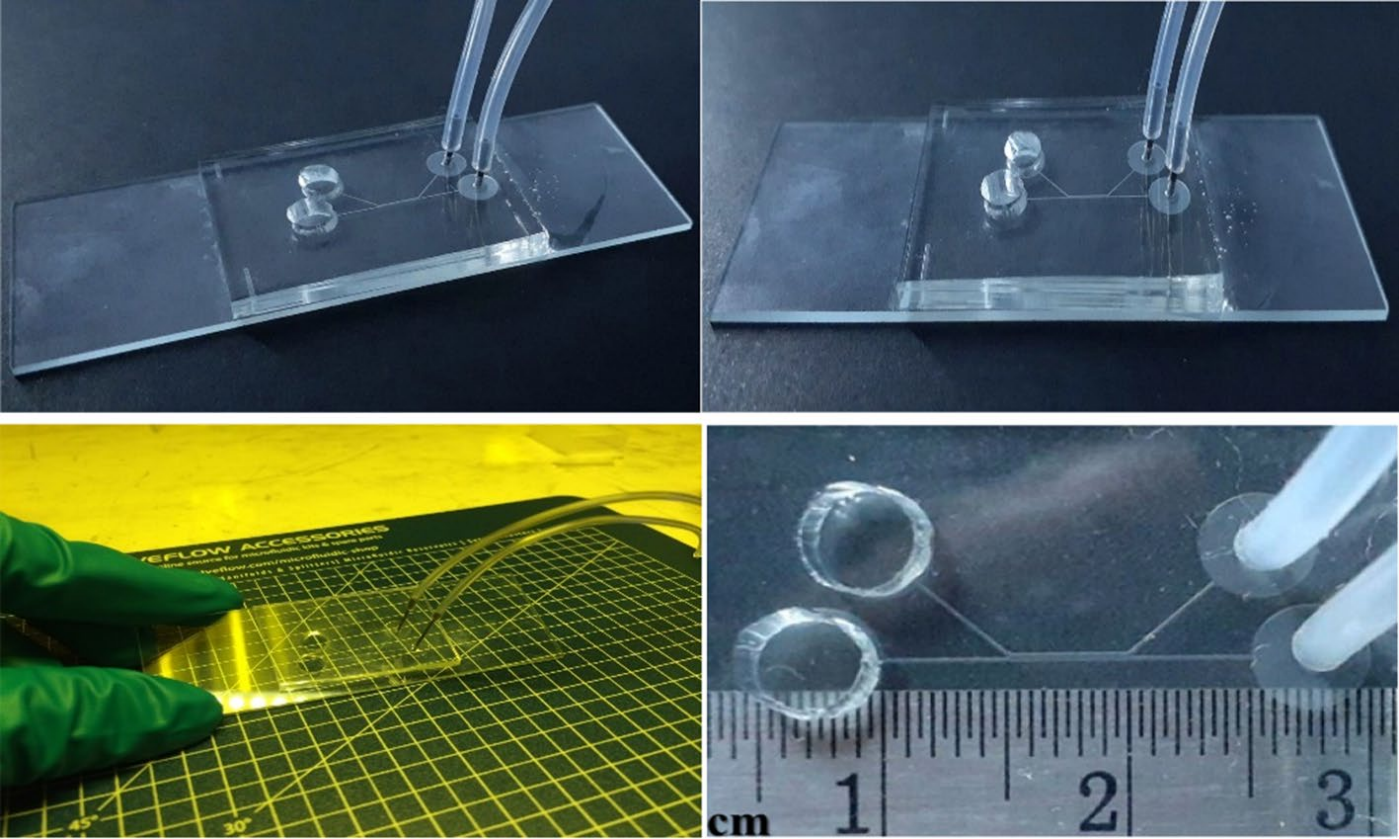
Microfluidic chip.

**Figure 9:**
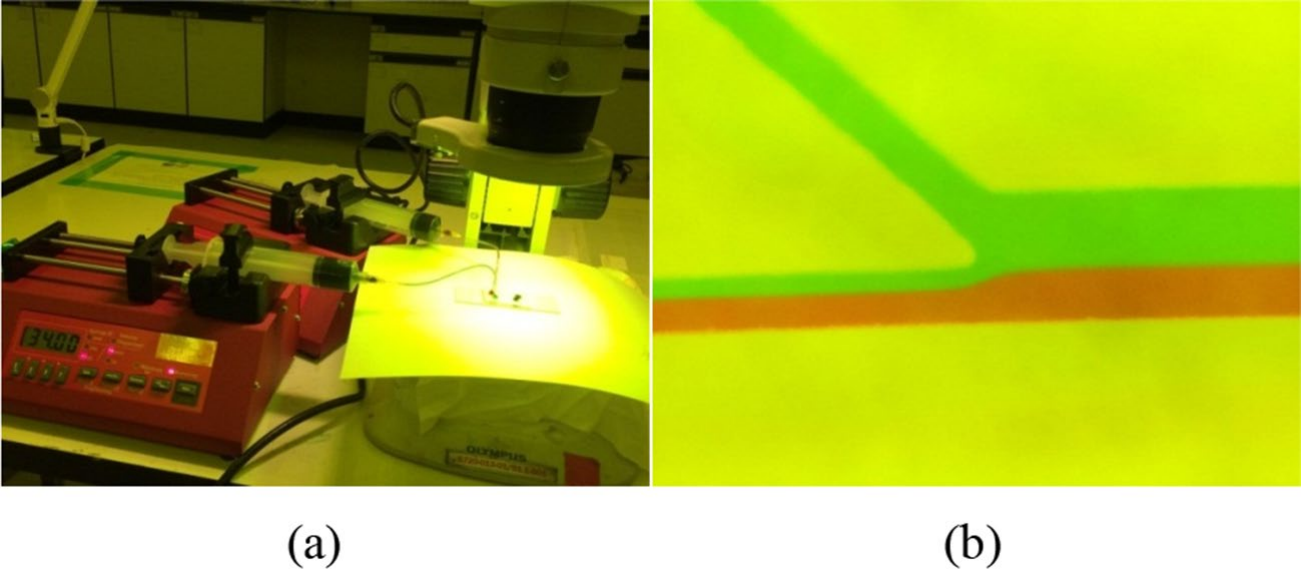
Sperm separation experiment by creeping flow at 40% of overall central channel width. (**a**) Experimental setup. (**b**) Creeping flow at 40% of overall central channel width for separation of motile sperm from nonmotile sperm.

A microfluidic chip (Fig. 8) was fabricated according to the output of the simulation program and used to investigate the optimized sperm cell separation device. The experimental setup is shown in Fig. 9a. One syringe pump controlled the sperm flow rate at the Channel SI inlet, and the other regulated the medium flow rate at the Channel MI inlet. Highly motile sperm swam upstream to Channel MSO, while nonmotile sperm exited via Channel NMSO, following the creeping flow model at 40% of the overall central or separation channel width. The experimental results were similar to those predicted by the simulation. The creeping flow at 40% of the overall central channel width enabled the structure to separate the motile and nonmotile sperm (Fig. 9b).

**Figure 10:**
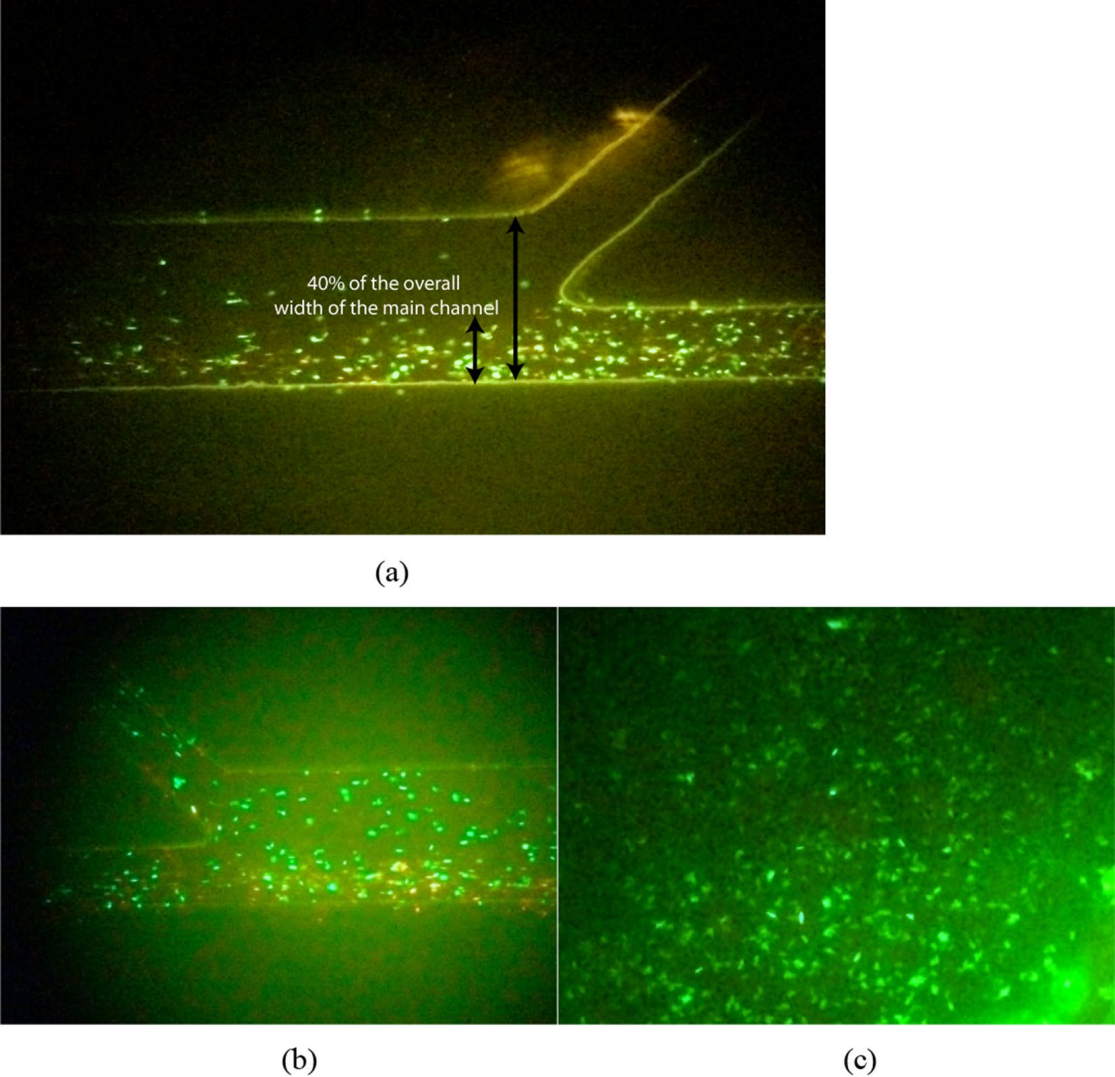
Sperm viability and fertilization potential. (**a**) Flow rate in microfluidic chip controlled by syringe pumps in Channels SI and MI at 0.24 µL min^−1^ and 0.34 µL min^−1^, respectively. (**b**) Sperm motility with healthy sperm crossing to Channel MSO. (**c**) Sperm motility and healthy sperm in Channel MSO.

**Figure 11:**
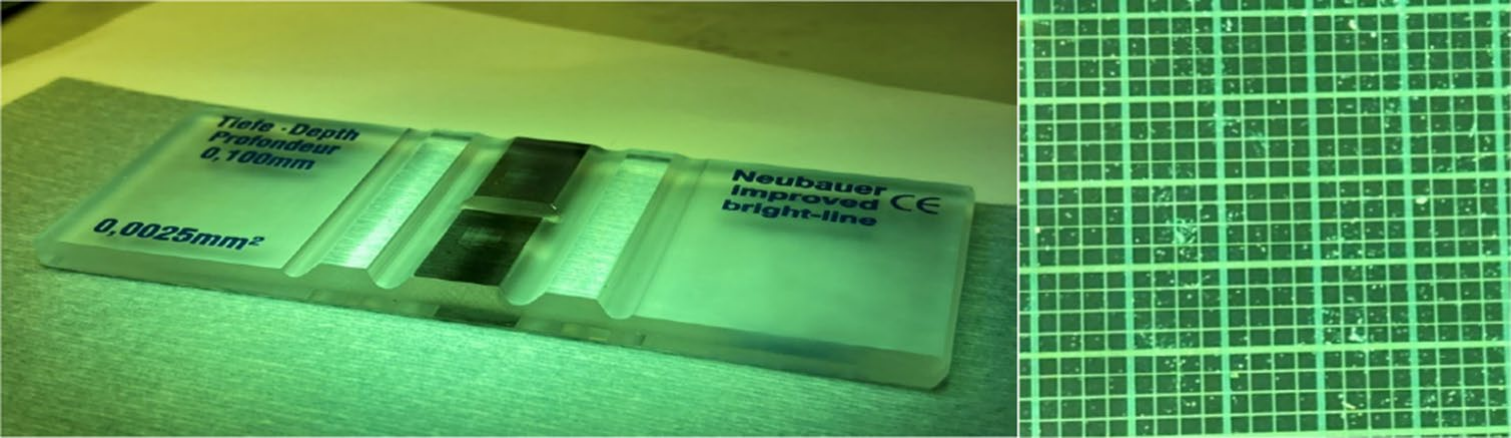
Hemocytometer.

The microfluidic chip was then tested by feeding bull sperm into it. The flow rate was controlled by a syringe pump connected to the sperm inlet Channel SI and the medium inlet Channel MI (Fig. 10a). Healthy motile sperm swam upstream to Channel MSO (Fig. 10b), and nonmotile/unhealthy motile sperm flowed to the Channel NMSO outlet. As shown in Fig. 10c (at 96.00% validation), sperm motility and health (6.94 × 10^5^ sperms counted) in Channel MSO could be noticed. By applying fluorescent dye and hemocytometer (Fig. 11), healthy motile/nonmotile sperm at MSO channel was counted. In five replicate experiments, this device separated motile and nonmotile sperm samples with an average 95.33% purity of ratio of motile sperms (Table 5). Based on this, we believe that our SSD performs approximately 5% better than the performance observed with existing methods.

**Table 5:** Sperm counts in Channel MSO after sperm passed through sorting device.

Motile sperm	Nonmotile sperm	% Purity
6.67 × 10^5^	2.78 × 10^4^	95.83
8.83 × 10^5^	5.56 × 10^4^	93.33
6.67 × 10^5^	2.78 × 10^4^	95.83
6.94 × 10^5^	2.78 × 10^4^	96.00
6.39 × 10^5^	2.78 × 10^4^	95.65
Average		95.33

### Confirmation of optimal values for sperm-sorting device

**Figure 12:**
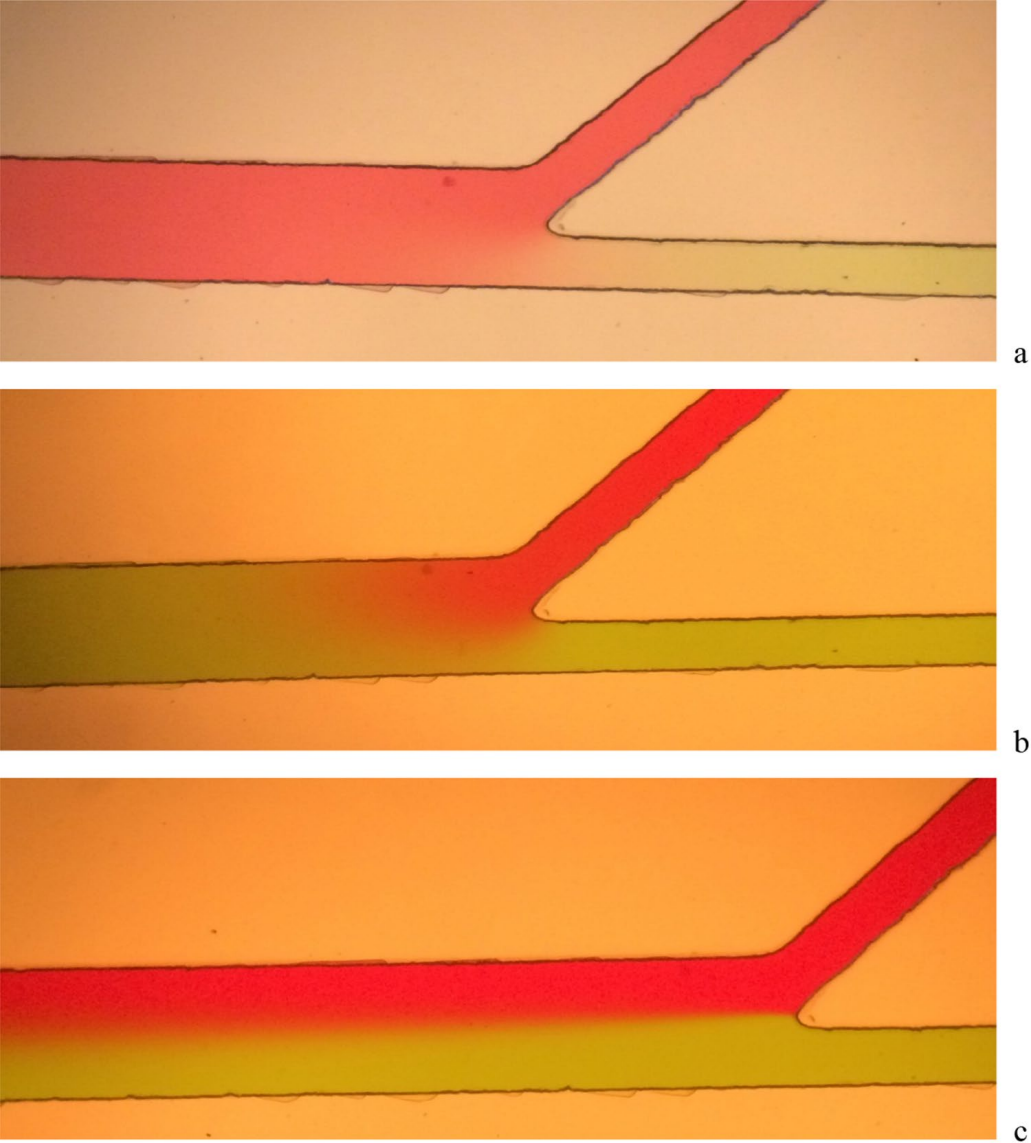
Fluid flow in microfluid chip (100 ×) (**a**) high medium inlet flow rate, (**b**) high sperm inlet flow rate, (**c**) sperm flow rate reached over 40% of the separation channel width.

The SSD is shown in Figs. 8 and 9. The experimental results confirmed that the parameter values determined from the DOE technique and the COMSOL simulation, i.e., angle between chambers = 43°, sperm/medium inlet and outlet chamber lengths = 5000 µm, separation chamber length = 5000 µm, separation chamber width = 400 µm, and sperm/medium inlet and outlet chamber widths = 200 µm, were optimal for the fabrication of the SSD. Soft lithography was used to fabricate a 50-µm thick PDMS-microchannel. Since this parameter did not affect our simulation range significantly, we fabricated at the highest thickness due to ease of fabrication. The syringe pump-impelled sperm and medium inlet flow rates were 0.24 µL min^−1^ and 0.34 µL min^−1^, respectively. The device output value was 40% of the overall central chamber width. The error between the optimal result and the target value was 0.02%. The error between the fabrication process result and the target value was 0.05%. The device precision was 97.28% (Table 6). Besides the optimal settings, we conducted the experiment in the optimal design microfluidic chip by changing medium and sperm flow rate. The images (Fig. 12a–c) were taken by using an inverted microscope (10 × Olympus Microscope IX71 with fluorescence and phase contrast). Figure 12a,b showing high medium and sperm flow rates, respectively, indicate that the lower fluid flow rate was dominated by the higher, which we was not desired. However, a reliable sorting device should have a flow as shown in Fig. 12c. These experiments also validated and promoted the potential of simulation and DOE technique for optimal chip design.

**Table 6:** Optimal values at 40% of overall central chamber width.

Response	Target	Optimization	Fabrication device	Optimal error (%)	Fabrication error (%)	Precision
40% of the overall central chamber width	40	40.02	40.5	0.02	0.047	97.28%

### Fluid viscosity range analysis

The fluid viscosity ranges were analyzed according to a COMSOL simulation. For a Newtonian fluid, viscosity ranges vary directly with dynamic viscosity. We investigated the fluid viscosity variations at constant sperm flow rate (0.24 µL min^−1^) and % of overall main channel width. Table 7 and Supplementary material Figs. A2–A5 show the simulation results for fluid viscosities of 0.00089 kg m^−1^ s^−1^, 0.000392 kg m^−1^ s^−1^, 0.00001 kg m^−1^ s^−1^, and 0.003 kg m^−1^ s^−1^.

**Table 7:** Simulation results of varied fluid viscosity.

Fluid viscosity at constant sperm flow rate (0.24 µL min^−1^) (kg m^−1^ s^−1^)	% of overall main channel width	Reynolds number	Fluid status
0.000890	40.00	7.00 × 10^–4^	Creeping flow
0.000392	40.00	4.244 × 10^–4^	Creeping flow
0.000010	42.20	2.26 × 10^3^	Turbulent flow
0.003000	38.89	7.546	Laminar flow

The simulation results (Table 7) revealed that the acceptable tolerance range for the fluid viscosity was 0.00001–0.003 kg m^−1^ s^−1^. At a constant flow rate, both high and low fluid viscosities elevated the Re. However, the % overall main channel width varied inversely with fluid viscosity. These responses could impair sorting device performance.

## Conclusions

In the present study, a sperm-sorting microfluidic device was designed and fabricated. The device structure was created by COMSOL simulation, and DOE was used to optimize the microfluidic chip for sorting of sperms. The DOE approach saves time and resources, and improves experimental efficiency. The microfluidic chip was fabricated according to the optimal values for the simulation output and performed effectively. Setting statistical significance at *p* < 0.05 facilitated analysis of the factors influencing the experimental design. At 97.99 desirability, the optimal microfluidic chip parameters were: (A) angle between chambers = 43°, (B) sperm inlet flow rate = 0.24 µL min^−1^, (C) medium inlet flow rate = 0.34 µL min^−1^, and (D) inlet and outlet chamber length = 5000 µm. The optimized values for the sperm-sorting factors were: (E) separation chamber length = 5000 µm, (F) chambers (inlet and outlet) thickness = 50 µm, (G) separation chamber width = 400 µm, and (H) chambers (inlet and outlet) width = 200 µm. There was good agreement between the simulation and experimental results, and the comparison accuracy was 97.28%. Accuracy of the microfluidic chip at sorting live and dead sperm was 95.33% on average.

The present study elucidated the major factors affecting sperm sorting. The experimental results identified the following most important parameters influencing the SSD structure: (A) angle between chambers, (B) sperm inlet flow rate, (C) medium inlet flow rate, and (D) inlet and outlet chamber lengths. The COMSOL program simulated the results. A comparison of the experimental and simulation model results indicated an accuracy close to 100%. The error was only 0.02% for the experiments executed to test the values from the simulation model results and to identify the optimal parameters. A comparison of the outputs of systematic and traditional designs indicated that the former generated a maximum value of 96%, while the latter provided maximum values of 95.24% and 92.16%^14^. We also performed fluid viscosity range analysis-based simulations and measured acceptable tolerances for the fluid viscosities in our SSD. We observed that the acceptable tolerance range for the fluid viscosity was 0.00001–0.003 kg m^−1^ s^−1^. Thus, our proposed microfluidic chip may be included in a sperm x/y separation device, which could benefit the livestock industry and as well as.

## Supplementary information

Supplementary Figures.

Supplementary Tables.
