# Supplementary material for: A novel microfluidic chip-based sperm-sorting device constructed using design of experiment method
Source: Sci Rep. 2020 Oct 13;10:17143. doi: 10.1038/s41598-020-73841-3 (PMC7553928; doi:10.1038/s41598-020-73841-3)
Supplement: Supplementary file 1 — Supplementary Figures. [file 41598_2020_73841_MOESM1_ESM.docx]

Supplementary A


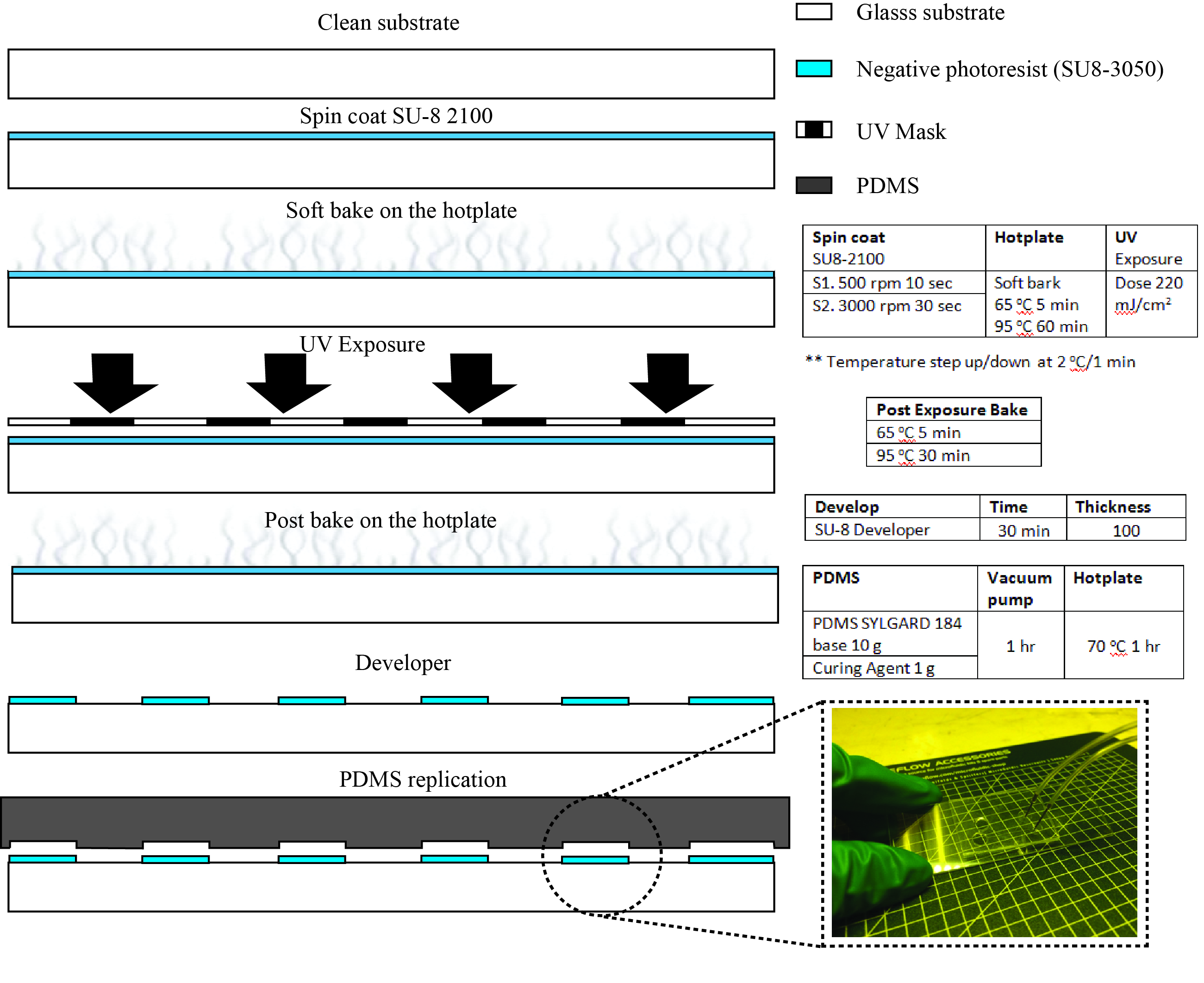


**Figure A1.** Microfluidic chip fabrication


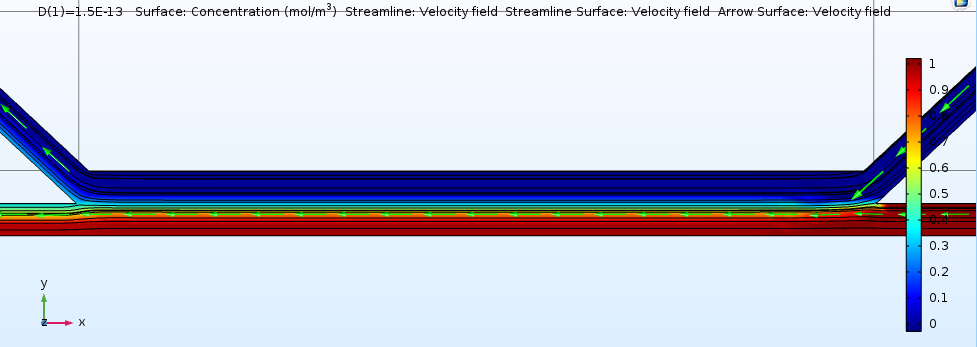


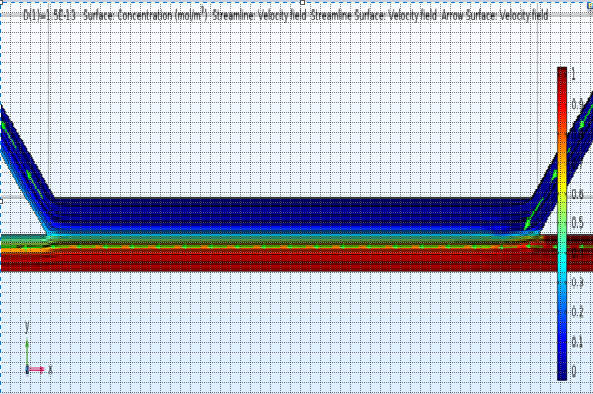


**Figure A2.** Simulation result at viscosity = 0.00089 kgm^-1^s^-1^ at flow rate = 0.24 ul/min


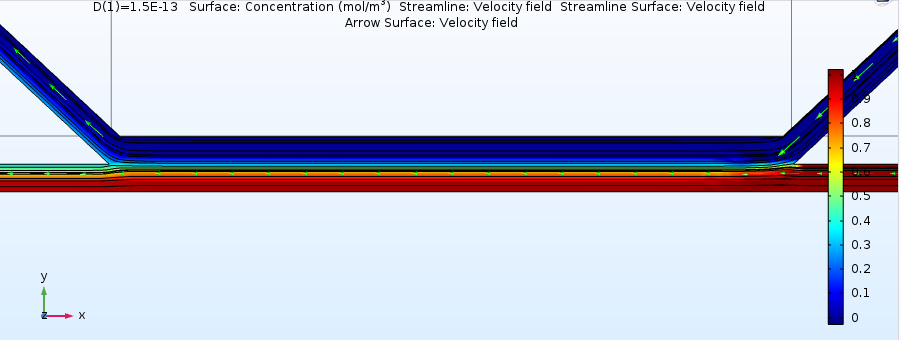


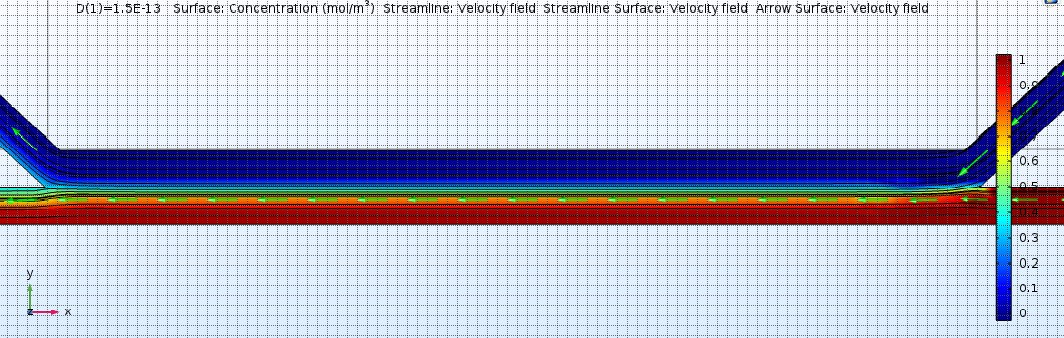


**Figure A3.** Simulation result at viscosity = 0.000392 kgm^-1^s^-1^ at flow rate = 0.24 ul/min


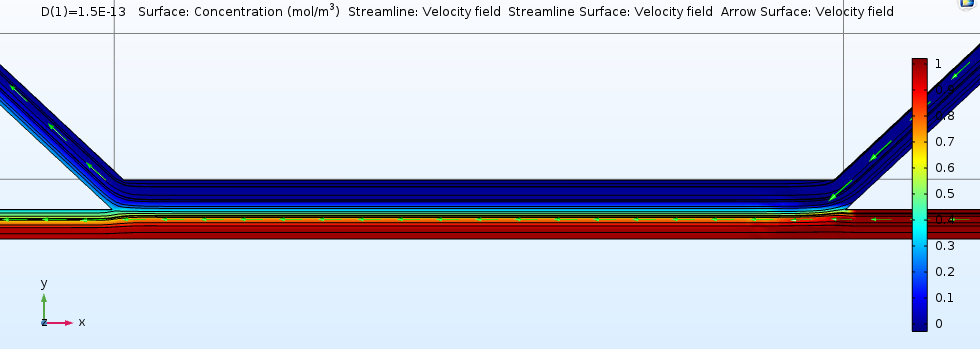


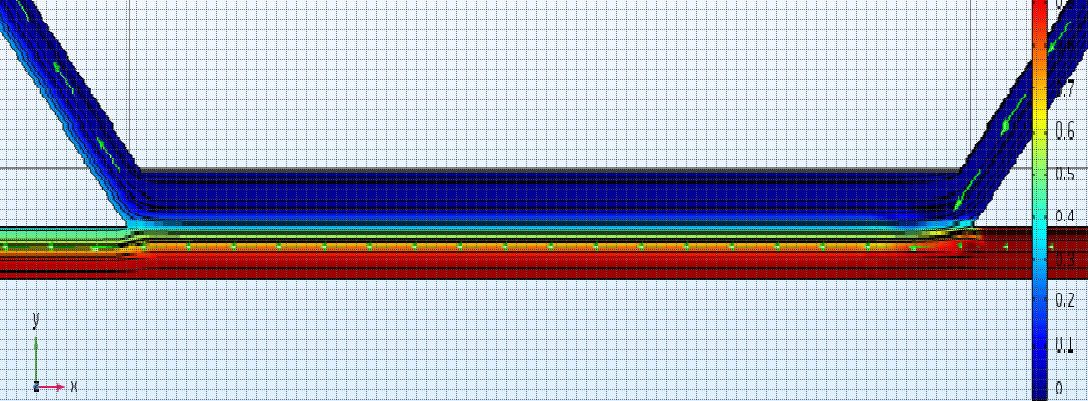


**Figure A4.** Simulation result at viscosity = 0.00001 kgm^-1^s^-1^ at flow rate = 0.24 ul/min


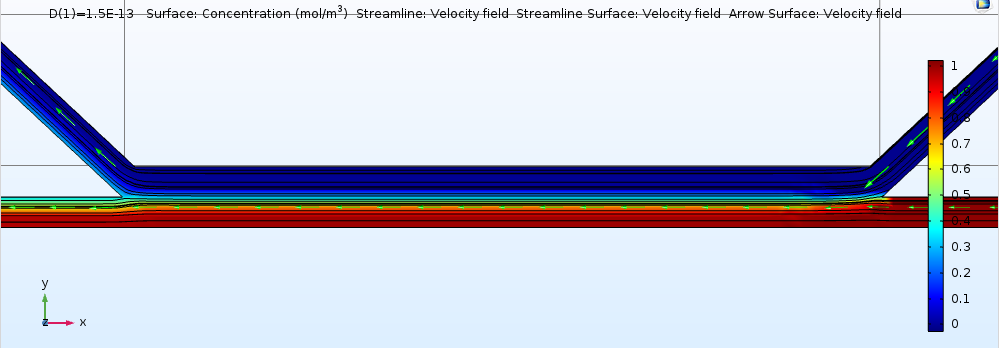


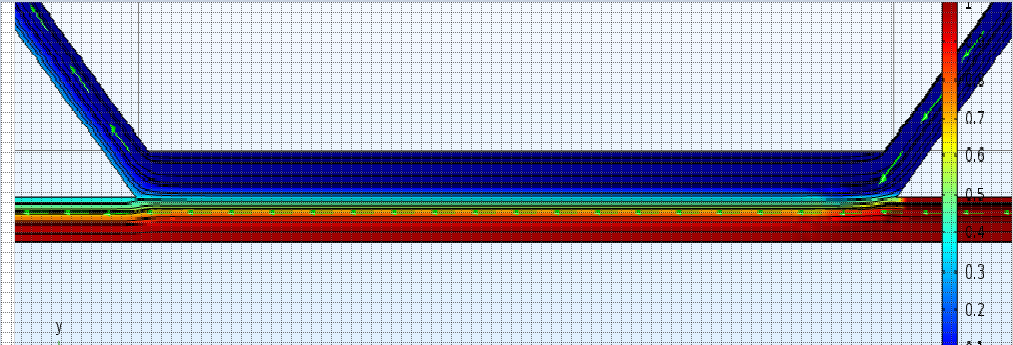


**Figure A5.** Simulation result at viscosity = 0.003 kgm^-1^s^-1^ at flow rate = 0.24 ul/min
