# Supplementary material for: A novel microfluidic chip-based sperm-sorting device constructed using design of experiment method
Source: Sci Rep. 2020 Oct 13;10:17143. doi: 10.1038/s41598-020-73841-3 (PMC7553928; doi:10.1038/s41598-020-73841-3)
Supplement: Supplementary file 2 — Supplementary Tables. [file 41598_2020_73841_MOESM2_ESM.docx]

Supplementary B

**Table B1** Result of 8 Factor: Response at 40% of the overall width of the central or separation channel versus A, B, C, D, E, F, G, and H

| **RunOrder** | **CenterPt** | **Blocks** | **A** | **B** | **C** | **D** | **E** | **F** | **G** | **H** | **Response 40%** |
| --- | --- | --- | --- | --- | --- | --- | --- | --- | --- | --- | --- |
| 1 | 1 | 1 | 43 | 0.4 | 0.2 | 3000 | 3000 | 50 | 400 | 100 | 48.23 |
| 2 | 1 | 1 | 33 | 0.4 | 0.45 | 5000 | 3000 | 30 | 300 | 100 | 50.23 |
| 3 | 1 | 1 | 33 | 0.4 | 0.45 | 3000 | 5000 | 30 | 400 | 200 | 42.22 |
| 4 | 1 | 1 | 43 | 0.4 | 0.45 | 3000 | 5000 | 50 | 300 | 200 | 40.12 |
| 5 | 1 | 1 | 33 | 0.15 | 0.2 | 3000 | 5000 | 30 | 400 | 100 | 52.34 |
| 6 | 1 | 1 | 43 | 0.4 | 0.2 | 3000 | 5000 | 50 | 400 | 200 | 54.25 |
| 7 | 1 | 1 | 43 | 0.15 | 0.2 | 3000 | 3000 | 50 | 300 | 200 | 43.60 |
| 8 | 1 | 1 | 33 | 0.15 | 0.45 | 3000 | 5000 | 30 | 300 | 100 | 40.34 |
| 9 | 1 | 1 | 33 | 0.15 | 0.2 | 5000 | 3000 | 50 | 300 | 100 | 50.35 |
| 10 | 1 | 1 | 33 | 0.4 | 0.2 | 5000 | 3000 | 50 | 400 | 200 | 63.81 |
| 11 | 1 | 1 | 33 | 0.4 | 0.2 | 5000 | 5000 | 30 | 400 | 200 | 64.62 |
| 12 | 1 | 1 | 33 | 0.15 | 0.45 | 5000 | 3000 | 50 | 400 | 100 | 44.13 |
| 13 | 1 | 1 | 43 | 0.15 | 0.2 | 5000 | 5000 | 50 | 400 | 100 | 45.45 |
| 14 | 1 | 1 | 43 | 0.4 | 0.45 | 5000 | 3000 | 50 | 400 | 100 | 43.14 |
| 15 | 1 | 1 | 33 | 0.15 | 0.2 | 3000 | 5000 | 50 | 400 | 200 | 48.89 |
| 16 | 1 | 1 | 33 | 0.4 | 0.2 | 3000 | 5000 | 30 | 300 | 200 | 60.12 |
| 17 | 1 | 1 | 33 | 0.15 | 0.45 | 3000 | 5000 | 50 | 300 | 200 | 42.27 |
| 18 | 1 | 1 | 33 | 0.15 | 0.45 | 5000 | 3000 | 30 | 400 | 200 | 45.15 |
| 19 | 1 | 1 | 43 | 0.15 | 0.2 | 5000 | 3000 | 50 | 400 | 200 | 47.37 |
| 20 | 1 | 1 | 33 | 0.4 | 0.2 | 3000 | 3000 | 30 | 300 | 100 | 61.82 |
| 21 | 1 | 1 | 33 | 0.4 | 0.2 | 5000 | 3000 | 30 | 400 | 100 | 60.03 |
| 22 | 1 | 1 | 43 | 0.15 | 0.45 | 5000 | 5000 | 30 | 300 | 200 | 28.10 |
| 23 | 1 | 1 | 43 | 0.15 | 0.2 | 3000 | 3000 | 30 | 300 | 100 | 42.02 |
| 24 | 1 | 1 | 43 | 0.15 | 0.45 | 5000 | 5000 | 50 | 300 | 100 | 29.85 |
| 25 | 1 | 1 | 33 | 0.15 | 0.45 | 3000 | 3000 | 50 | 300 | 100 | 40.34 |
| 26 | 1 | 1 | 43 | 0.4 | 0.2 | 5000 | 3000 | 30 | 300 | 200 | 59.71 |
| 27 | 1 | 1 | 43 | 0.15 | 0.2 | 5000 | 3000 | 30 | 400 | 100 | 44.86 |
| 28 | 1 | 1 | 33 | 0.15 | 0.2 | 3000 | 3000 | 50 | 400 | 100 | 51.45 |
| 29 | 1 | 1 | 33 | 0.4 | 0.2 | 3000 | 5000 | 50 | 300 | 100 | 61.69 |
| 30 | 1 | 1 | 33 | 0.4 | 0.45 | 3000 | 5000 | 50 | 400 | 100 | 47.08 |
| 31 | 1 | 1 | 43 | 0.15 | 0.2 | 3000 | 5000 | 50 | 300 | 100 | 41.46 |
| 32 | 1 | 1 | 43 | 0.15 | 0.45 | 3000 | 3000 | 30 | 400 | 100 | 27.36 |
| 33 | 1 | 1 | 33 | 0.15 | 0.45 | 5000 | 5000 | 50 | 400 | 200 | 45.45 |
| 34 | 1 | 1 | 43 | 0.15 | 0.45 | 3000 | 5000 | 50 | 400 | 100 | 26.48 |
| 35 | 1 | 1 | 33 | 0.15 | 0.2 | 5000 | 5000 | 50 | 300 | 200 | 53.94 |
| 36 | 1 | 1 | 43 | 0.4 | 0.2 | 3000 | 3000 | 30 | 400 | 200 | 59.11 |
| 37 | 1 | 1 | 33 | 0.4 | 0.45 | 5000 | 3000 | 50 | 300 | 200 | 49.44 |
| 38 | 1 | 1 | 43 | 0.15 | 0.45 | 5000 | 3000 | 30 | 300 | 100 | 30.22 |
| 39 | 1 | 1 | 43 | 0.4 | 0.45 | 5000 | 3000 | 30 | 400 | 200 | 45.92 |
| 40 | 1 | 1 | 43 | 0.4 | 0.45 | 5000 | 5000 | 50 | 400 | 200 | 44.00 |
| 41 | 1 | 1 | 33 | 0.4 | 0.45 | 3000 | 3000 | 30 | 400 | 100 | 47.83 |
| 42 | 1 | 1 | 43 | 0.4 | 0.45 | 5000 | 5000 | 30 | 400 | 100 | 44.78 |
| 43 | 1 | 1 | 33 | 0.4 | 0.45 | 5000 | 5000 | 30 | 300 | 200 | 52.48 |
| 44 | 1 | 1 | 33 | 0.15 | 0.2 | 3000 | 3000 | 30 | 400 | 200 | 49.10 |
| 45 | 1 | 1 | 43 | 0.15 | 0.45 | 3000 | 3000 | 50 | 400 | 200 | 27.58 |
| 46 | 1 | 1 | 43 | 0.4 | 0.45 | 3000 | 5000 | 30 | 300 | 100 | 40.44 |
| 47 | 1 | 1 | 43 | 0.4 | 0.45 | 3000 | 3000 | 50 | 300 | 100 | 42.50 |
| 48 | 1 | 1 | 33 | 0.15 | 0.2 | 5000 | 3000 | 30 | 300 | 200 | 51.32 |
| 49 | 1 | 1 | 33 | 0.4 | 0.2 | 3000 | 3000 | 50 | 300 | 200 | 62.50 |
| 50 | 1 | 1 | 43 | 0.4 | 0.2 | 5000 | 3000 | 50 | 300 | 100 | 58.29 |
| 51 | 1 | 1 | 33 | 0.4 | 0.45 | 3000 | 3000 | 50 | 400 | 200 | 48.53 |
| 52 | 1 | 1 | 43 | 0.15 | 0.2 | 5000 | 5000 | 30 | 400 | 200 | 44.74 |
| 53 | 1 | 1 | 33 | 0.4 | 0.45 | 5000 | 5000 | 50 | 300 | 100 | 51.50 |
| 54 | 1 | 1 | 43 | 0.4 | 0.2 | 3000 | 5000 | 30 | 400 | 100 | 59.64 |
| 55 | 1 | 1 | 43 | 0.15 | 0.45 | 5000 | 3000 | 50 | 300 | 200 | 30.33 |
| 56 | 1 | 1 | 43 | 0.15 | 0.45 | 3000 | 5000 | 30 | 400 | 200 | 25.48 |
| 57 | 1 | 1 | 33 | 0.15 | 0.45 | 3000 | 3000 | 30 | 300 | 200 | 43.12 |
| 58 | 1 | 1 | 43 | 0.4 | 0.2 | 5000 | 5000 | 30 | 300 | 100 | 60.00 |
| 59 | 1 | 1 | 43 | 0.4 | 0.45 | 3000 | 3000 | 30 | 300 | 200 | 42.86 |
| 60 | 1 | 1 | 33 | 0.4 | 0.2 | 5000 | 5000 | 50 | 400 | 100 | 62.33 |
| 61 | 1 | 1 | 33 | 0.15 | 0.2 | 5000 | 5000 | 30 | 300 | 100 | 49.53 |
| 62 | 1 | 1 | 33 | 0.15 | 0.45 | 5000 | 5000 | 30 | 400 | 100 | 44.92 |
| 63 | 1 | 1 | 43 | 0.4 | 0.2 | 5000 | 5000 | 50 | 300 | 200 | 54.49 |
| 64 | 1 | 1 | 43 | 0.15 | 0.2 | 3000 | 5000 | 30 | 300 | 200 | 40.54 |
